# The Ballet of Natural-Product: Carrier-Free “Triadic” Drug Delivery Platforms for Enhanced Tumor Treatment

**DOI:** 10.3390/jfb16120433

**Published:** 2025-11-25

**Authors:** Liyan Yang, Zhonglei Wang

**Affiliations:** 1 School of Physics and Physical Engineering, Qufu Normal University, Qufu 273165, China; 2 Beijing National Laboratory for Molecular Sciences, Institute of Chemistry, Chinese Academy of Sciences, Beijing 100190, China; 3 Key Laboratory of Green Natural Products and Pharmaceutical Intermediates in Colleges and Universities of Shandong Province, School of Chemistry and Chemical Engineering, Qufu Normal University, Qufu 273165, China; 4 Key Laboratory of Bioorganic Phosphorous Chemistry and Chemical Biology, School of Pharmaceutical Sciences, Tsinghua University, Beijing 100084, China

**Keywords:** cancer, natural products, nanomedicine, carrier-free drug delivery platforms, “triadic” strategies, multidrug resistance

## Abstract

Cancer poses a considerable challenge to global public health and stands as the second leading cause of mortality worldwide. Chemotherapy provides limited benefits for advanced-stage cancer, mainly due to high systemic toxicity and drug resistance. Optimal cancer treatment requires a sophisticated, multidisciplinary collaboration aimed at extending survival, enhancing quality of life, and reducing toxicity. Natural products present advantages, including a wide array of structural diversity, reduced toxicity, improved immune modulation, and the ability to act on multiple targets. Nanomedicine design shows promise in tumor treatment and diagnosis by improving efficacy and minimizing side effects. Due to the heterogeneity of tumors in genetics, metabolism, and microenvironment, natural product-based carrier-free drug delivery platforms have been actively investigated and demonstrated considerable potential for enhanced tumor treatment. “Triadic” strategies can simultaneously perform various functions on a carrier-free intelligent nanoplatform. These include combinational chemotherapy, photodynamic therapy (PDT) with bioimaging and chemotherapy, PDT combined with photothermal therapy (PTT) and chemotherapy, chemo-radio-theranostics, as well as gene therapy (GT) in conjunction with PTT and chemotherapy. This multifaceted approach enhances therapeutic efficacy, reduces multidrug resistance, and minimizes systemic toxicity. This review encompasses recent advancements in cancer therapy using carrier-free “triadic” nanomedicines based on natural products (between 2024 and 2025) and evaluates this evolving field, emphasizing the pivotal role of natural products—berberine, camptothecin, hypericin, erianin, curcumin, lactose, paclitaxel, gambogic acid, and glycyrrhizic acid—in drug delivery platforms. Furthermore, it addresses the challenges and bottlenecks encountered by carrier-free drug delivery platforms, offering valuable insights into their development trajectories.

## 1. Introduction

**Figure 1:**
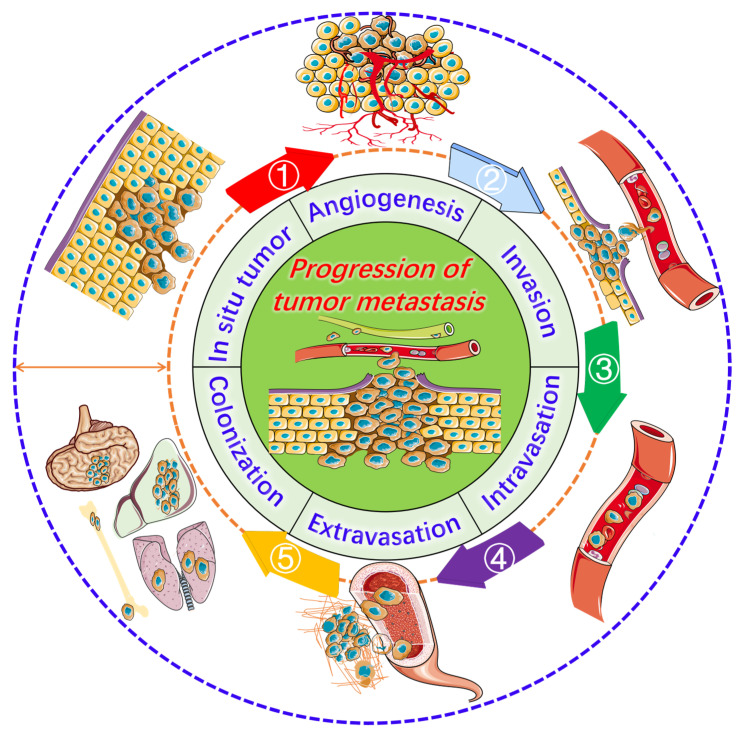
Progression of tumor metastasis. The process starts with an in situ tumor, leading to angiogenesis and the activation of invasion, intravasation, extravasation, and ultimately metastasis to the lungs, liver, brain, and bones.

Cancer is the second leading cause of death [1]. It is a malignant disease characterized by invasiveness and metastasis and is caused by genetic and epigenetic alterations [2,3]. These changes lead to abnormal cell cycle regulation, resistance to apoptosis, unlimited replication, metabolic reprogramming, and genomic instability [4,5]. Unlike benign tumors, malignant tumors can invade blood vessels and lymphatic systems, metastasizing to distant sites and damaging organ function (Figure 1) while posing a global health threat [6,7]. Multidisciplinary treatment based on individualization and integration is essential for improving prognosis [8,9]. Surgical resection [10] remains crucial for the radical treatment of localized malignant tumors, with radiotherapy [11] and immunotherapy [12,13] often used as adjuvant or concurrent therapies. Targeted therapy (focusing on specific driver genes or pathways) [14] has significantly enhanced survival rates across various cancers, and it is crucial for clinical cancer treatment. The development of combined treatment strategies [15] and individualized regimens [16] is currently a key focus area. Additionally, emerging technologies like GT [17], RNA intervention [18], and nanodrug delivery [19,20,21] are progressing toward clinical application; however, challenges such as safety concerns [22], multidrug resistance (MDR) [23,24], and accessibility [25] must be addressed. Maximizing therapeutic efficacy while minimizing toxicity remains the core challenge in clinical practice.

**Figure 2:**
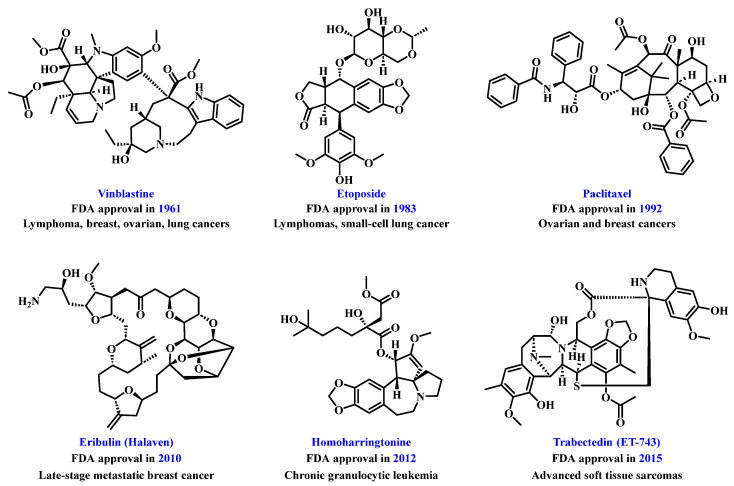
Chemical structures of representative natural product-based anti-tumor drugs.

Natural products are a vital source of inspiration for drug development, significantly contributing to human health [26,27,28,29,30,31,32,33,34,35]. Their role in tumor treatment has a long history, with marine organisms [36], traditional medicinal plants [37,38,39], and microbial metabolites [40] offering rich chemical diversity for drug discovery. Research on natural product-derived anti-tumor agents has become a key focus in medicine [41,42]. This has led to the emergence of various effective and somewhat selective anti-tumor compounds, such as the U.S. Food and Drug Administration (FDA)-approved paclitaxel, podophyllotoxin, homoharringtonine, vinblastine, eribulin, and ET-743 (Figure 2). However, challenges remain in applying natural products for tumor treatment due to poor solubility [43,44] and suboptimal pharmacokinetics [45,46]. Researchers have developed strategies such as structural modification [47], combination therapy [48,49], prodrug strategies [50,51], and advanced drug delivery systems [52,53] to improve bioavailability and targeting. Technologies like polymer nanoparticles (NPs) [54], liposomes [55], extracellular vesicles [56], and carrier-free drug delivery platforms [57] are enhancing the therapeutic efficacy of natural products.

**Figure 3:**
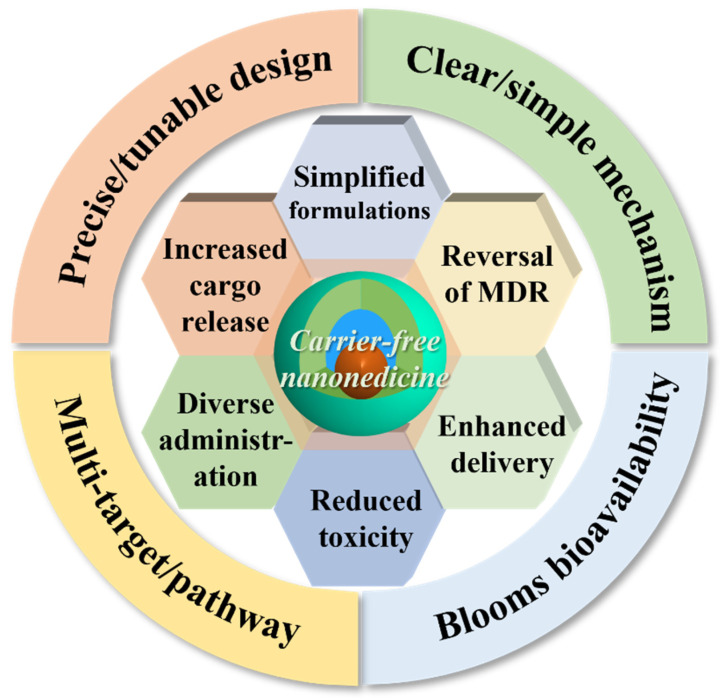
The advantages of carrier-free drug delivery platforms.

Nevertheless, the materials used for delivering nanomedicines contribute to increased preparation costs, diminished controllability, and more complex processes [54]. Additionally, nanocarriers often exhibit low drug-loading capacities, making it challenging to ensure encapsulation efficiency [55]. Furthermore, the impact of nanocarriers on biological systems—such as immunogenicity and potential toxicity—requires thorough evaluation due to possible adverse effects stemming from their degradation and metabolism [56]. Consequently, there is an urgent need for carrier-free nanomedicines [57]. In comparison to traditional nanomedicines, the carrier-free drug delivery platform fundamentally disrupts traditional models: the payload itself serves as the transport vehicle, which can either be a drug scaffold formed through covalent connections (e.g., glutathione (GSH)-responsive disulfide bond and reactive oxygen species (ROS)-responsive thioacetone bond) or a self-assembled structure driven by the intrinsic physicochemical properties of drugs (including π-π stacking, hydrophobic interactions, and electrostatic interactions) [58]. The potential advantages (Figure 3) include nearly complete drug-loading capacity, reduced toxicity associated with carriers, enhanced biosafety, improved cost-effectiveness, simplified regulatory pathways for certain constructs, and the possibility of synchronized pharmacokinetics in combination therapies.

Natural product-based carrier-free nanomedicines are emerging as a promising approach for individualized treatment [59,60]. The key idea is to use bioactive natural products as building blocks, forming NPs with diameters of 1 to 200 nm through self-assembly, drug-drug covalent derivatization, or simple physical mixing, without traditional carriers [61,62]. This strategy aims to enhance drug loading, reduce carrier-related toxicity, simplify preparation steps, improved pharmacokinetics, and improve drug effectiveness in the tumor microenvironment (TME) [63,64]. Design principles and implementation strategies are crucial in this field. Common methods involve: (1) Drugs self-aggregating into NPs driven by their hydrophobicity and intermolecular interactions. (2) Drug-drug covalent derivatization creating degradable prodrugs that release active drugs under specific tumor conditions. (3) Fine-tuning size and surface charge using trace auxiliary molecules while avoiding conventional carriers. (4) Natural products interacting with other assembly factors to form multi-drug composite nano-systems that exhibit synergistic anti-tumor effects. The goal is to achieve stable particle size distribution, prolonged blood circulation time, and targeted accumulation in tumors. By eliminating carrier materials, this approach may reduce long-term accumulation issues and enhance patient tolerance and therapeutic efficacy [65].

**Figure 4:**
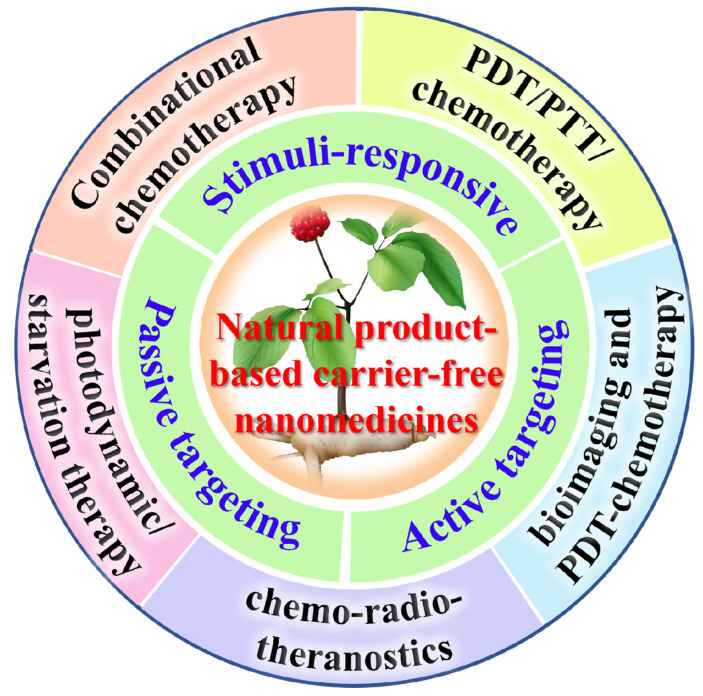
Natural product-based “triadic” carrier-free nanomedicines for tumor treatment.

Natural products such as polyphenols [66,67], triterpenoids [68], alkaloids [69,70], and flavonoids [71] exhibit strong hydrophobicity and aromatic ring structures, making them prone to aggregation in aqueous. Under specific conditions, they can self-assemble into NPs, enhancing water solubility and bioavailability [72,73]. Controllable assembly and drug release can be achieved through chemical modifications, optimizing the hydrophilic/hydrophobic balance, and managing dissociation equilibrium [74,75]. Currently, an increasing number of studies are focusing on the combined application of natural products with chemotherapy, immunotherapy, photodynamic therapy (PDT), bioimaging, photothermal therapy (PTT), gene therapy (GT), and radiotherapy, exploring new strategies (through passive targeting, active targeting, and stimuli-responsive targeting) for synergistic enhancement and drug resistance overcoming [76,77,78,79,80,81,82] (Table 1, Figure 4). The “triadic” drug delivery system enables innovative multi-dimensional therapies to effectively combat various forms of drug resistance.

This review explores natural product-derived, carrier-free, multimodal drug delivery strategies (between 2024 and 2025) and aims to inspire innovative ideas for cancer therapy protocols. Major digital databases, including Web of Science, PubMed, and Google Scholar, were perused using a series of search terms: natural products, carrier-free, nanomedicines, nanoparticles, nanoplatform, multimodal cancer therapy, PDT, PTT, GT, theranostics, and MDR. We believe this work effectively bridges recent advances in nanomedicine design with the biological advantages of natural compounds, offering valuable insights for future clinical translation.

## 2. Natural Product-Based Carrier-Free Nanomedicines for Cancer Combinational Chemotherapy

As early as the 1960s, combined chemotherapy—utilizing two or more cytotoxic or targeted drugs simultaneously—showed significant efficacy in preclinical models and achieved clinical success in various cancer treatments [83,84]. Early studies confirmed that multidrug strategies were more effective at controlling tumors than single-drug therapies, a concept that has dominated oncology for decades [85]. In response to the diversity of cancer biology, tumor burden, patient conditions, and treatment goals, numerous regimens have been developed over time. The rational design of combined therapy is guided by principles aimed at maximizing anti-tumor activity while minimizing toxicity. The four core principles—minimizing toxic overlap; employing different mechanisms of action to avoid cross-resistance; optimizing dose ratios for synergistic effects; and ensuring compatibility of solubility and permeability for effective delivery—provide a solid framework for designing and evaluating multidrug therapy regimens.

**Figure 5:**
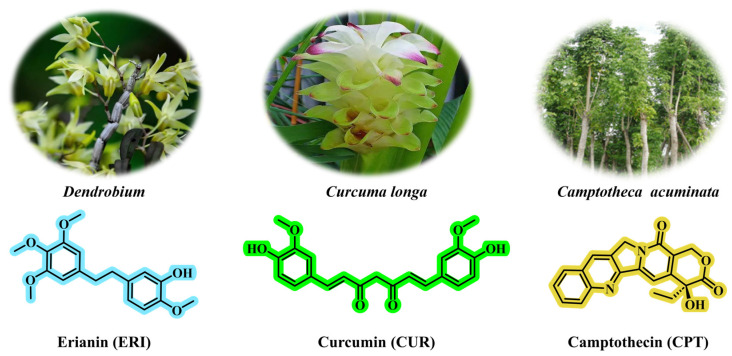
The chemical structures of erianin, curcumin, and camptothecin. Erianin can be isolated from the medicinal plant *Dendrobium*; curcumin can be isolated from *Curcuma longa*; camptothecin can be isolated from *Camptotheca acuminata*.

Prodrugs are ingenious derivatives of therapeutic agents, designed to improve the pharmacokinetic properties of the drugs [86]. In the field of combined chemotherapy, the use of active anticancer drugs as structural components in prodrug design has rapidly advanced [87]. This approach enables high drug loading, improved reproducibility, and consistent pharmacokinetic, and pharmacodynamic profiles. Instead of merely combining different drugs, it integrates the active drug into a single molecular framework or coordinates it with prodrugs for synergistic delivery and controlled release, allowing tumor cells to encounter cytotoxic or targeted agents simultaneously [88]. This strategy employs two main methods: (i) attaching drugs to a single chain backbone to create multi-component conjugates; (ii) linking agents as independent prodrug units to form customizable families with tailored release characteristics. These designs enhance potential drug loading while maintaining or improving therapeutic specificity and clinical feasibility. Prodrug-based multidrug combinations aim for precise delivery by controlling exposure levels, enhancing the therapeutic index, potentially reducing peak-related toxicity, and minimizing non-target effects [89,90]. In preclinical and early clinical studies, these systems have demonstrated stronger tumor selectivity and more consistent pharmacokinetics among carriers; in some cases, their anti-tumor efficacy exceeds that of traditional combination therapies. This design overcomes limitations of conventional drug delivery systems and offers new strategies for cancer treatment. Erianin (ERI), a compound derived from traditional Chinese medicine *Dendrobium*, exhibits significant anticancer properties through various molecular mechanisms. Notably, ERI inhibits the growth and migration of lung cancer cells by inducing Ca^2+^/CaM-dependent ferroptosis [76]. (Figure 5). Curcumin (CUR), derived from the vibrant turmeric plant (*Curcuma longa*), demonstrates remarkable anticancer properties (Figure 5) [3]. Camptothecin (CPT), a topoisomerase I inhibitor derived from *Camptotheca acuminata*, is an effective anticancer agent that demonstrates significant anti-tumor activity against various types of cancer (Figure 5) [15].

**Figure 6:**
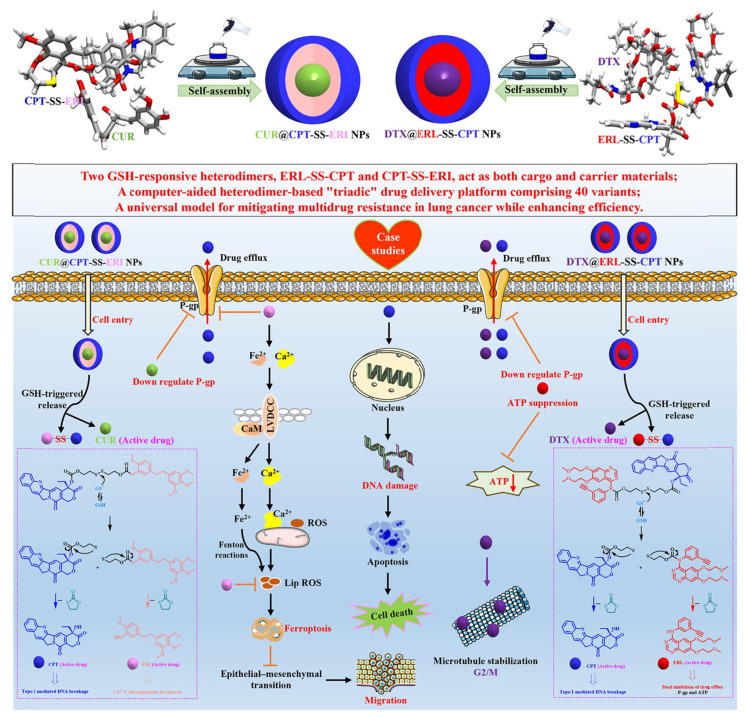
Schematic illustration depicting the preparation and synergistic chemotherapy of the “triadic” drug delivery platform. Reproduced from Ref. [76] with permission from © 2024 Elsevier Inc.

Motivated by a multi-modality therapeutic strategy, we designed a universal “3-in-1” self-delivery system using computer-aided methods [76]. This system effectively alleviated MDR in lung cancer and significantly enhanced therapeutic efficiency by integrating two GSH-responsive heterodimers (ERL-SS-CPT and CPT-SS-ERI; Figure 6). These heterodimers served as both drug carriers and the drugs themselves, forming NPs through self-assembly. The average hydrodynamic diameters were precisely controlled at 94.0 nm and 83.1 nm, respectively, ensuring effective drug delivery. The optimal size distribution was achieved at a 1:1 molar ratio (approximately 108 nm for CUR@CPT-SS-ERI NPs and 118 nm for DTX@ERL-SS-CPT NPs). This result significantly improved the efficiency and stability of co-delivery systems. A 20-day storage stability test at 4 °C showed that CPT-SS-ERI NPs, CUR@CPT-SS-ERI NPs, ERL-SS-CPT NPs, and DTX@ERL-SS-CPT NPs maintained good particle size distribution and morphology without significant aggregation or precipitation, demonstrating excellent physical stability. This study evaluated the chemical stability of NPs in biological environments.

Research on the GSH-responsive drug release mechanism indicated that NPs remained stable under normal physiological conditions (low GSH concentration) while precisely triggering drug release in high-GSH environments typical of tumor cells. This intelligent responsiveness enhanced both effectiveness and safety. Combining results from particle size control and stability assessments, the “three-in-one” NPs exhibited outstanding physical and chemical properties: a high drug-loading rate (over 76%), good dispersion, and negative surface charge to enhance circulation time. Additionally, it revealed insights into its self-assembly mechanism through multi-scale simulations. Utilizing computer-aided design methods alongside molecular dynamics simulations allowed for an in-depth exploration of the molecular mechanisms behind heterodimer self-assembly.

This interdisciplinary research method established a scientific foundation for the rational design of nanomedicine delivery systems. Utilizing the self-assembly properties of heterodimers, a versatile “three-in-one” drug delivery platform with 40 variants was successfully developed. This platform enabled flexible loading of various drug combinations by adjusting heterodimer types and proportions, highlighting high modularity and customizability to support personalized medicine. By incorporating GSH-responsive disulfide bond linkers, precise drug release from NPs in the high-GSH tumor cells was achieved. The study facilitated multimodal synergistic treatment of lung cancer cells through co-loading multiple drugs (e.g., DTX@ERL-SS-CPT NPs and CUR@CPT-SS-ERI NPs). Through detailed in vitro experiments, we thoroughly evaluated the physical and chemical properties, storage stability, GSH-responsive drug release, cellular uptake, apoptotic effects, biocompatibility, and cytotoxicity of the developed NPs. Additionally, we delved into the molecular mechanisms underlying the overcoming of MDR. These systematic investigations not only advanced technological progress in drug delivery systems but also provided new effective strategies for treating lung cancer and malignant tumors. Take CUR@CPT-SS-ERI NPs as an illustrative example. CUR downregulates P-glycoprotein (P-gp) in cancer cells by inhibiting the PI3K/Akt/NF-kB signaling pathway, thereby enhancing the anti-tumor efficacy of CPT. ERI inhibits the growth and migration of lung cancer cells through Ca^2+^/calmodulin-dependent ferroptosis and significantly reduces P-gp expression, effectively modulating the MDR phenotype in oxaliplatin-resistant cells. Furthermore, CPT exerts its effects by inhibiting DNA Topoisomerase I, leading to DNA damage in cancer cells. We believe that this work provides valuable insights for future clinical applications.

This platform significantly reduced MDR and enhanced anti-tumor efficiency. Notably, the in vitro experiments and some mechanism studies are presented, but there is a lack of data from preclinical or clinical trials. Including animal experiment data, particularly long-term toxicity, and pharmacodynamic studies, as well as preliminary clinical trial results, would enhance the research’s credibility and practicality. While the self-assembly mechanism of heterodimers and drug release mechanisms were discussed, specific molecular interactions and signaling pathways were not thoroughly analyzed. The choice of drug combinations may influence therapeutic effects, yet the research does not adequately compare the impact of different combinations.

## 3. Natural Product-Based Carrier-Free Nanoparticles for Liver Cancer PDT/PTT/Chemotherapy

PDT and PTT are emerging techniques for tumor ablation that utilize light activation to selectively destroy cancer cells, enhancing minimally invasive oncology with significant potential for combined applications [91,92]. PDT uses a photosensitizer (PS) that accumulates in tumor tissues and is activated by specific wavelength light [93]. In the presence of oxygen, the activated PS generates ROS, particularly singlet oxygen, which induce oxidative damage to cellular components, leading to the death of tumor cells through apoptosis, necrosis, or autophagy [94]. However, PDT faces challenges such as optimizing tumor selectivity, reducing long-term photosensitivity effects, and addressing uneven tumor oxygenation [95]. When exposed to near-infrared light (650 to 1100 nm), these agents increase local temperatures causing coagulative necrosis, rupture of cell membranes, protein denaturation, and ultimately cell death [96].

The theory of “medicine and food homology” has a long history in China and has always emphasized the concept of “maintaining health through food and treating diseases through medicine” [97,98,99,100,101,102]. Many traditional Chinese medicinal materials can be used not only as drugs for treatment but also as daily food ingredients for human consumption, demonstrating the interchangeability and complementarity between drugs and food [103,104,105,106,107,108,109,110]. Modern research has further revealed the molecular basis behind this: these medicinal materials are generally rich in active components such as amino acids [111], flavonoids [112], and terpenoids [113], and possess multiple biological effects including antioxidation [114], anti-tumor [115], and immune regulation [116]. Take licorice as an example, its chemical components include flavonoids, GL, and related glycosides [117,118]. It not only plays a role in drug treatment but can also be consumed through the daily diet to exert health care functions such as alleviating inflammation and protecting mucous membranes [119,120]. These components may achieve a synergistic effect through multi-target mechanisms, becoming an essential material basis for “medicine and food homology” [121].

**Figure 7:**
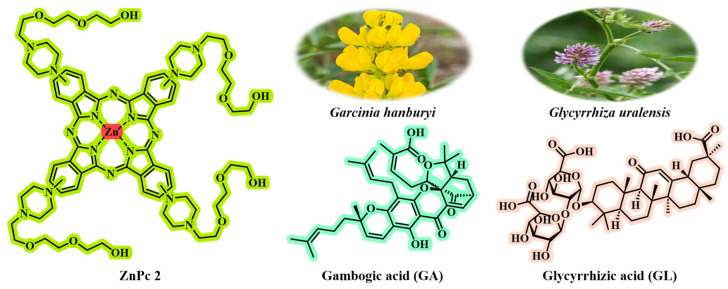
The chemical structures of ZnPc_2_, gambogic acid, and glycyrrhizic acid. Gambogic acid can be isolated from the medicinal plant *G. hanburyi*; glycyrrhizic acid can be isolated from *Glycyrrhiza uralensis*.

GL, a food-grade saponin with excellent interfacial activity, has liver-targeting properties (specifically recognizing overexpressed GL receptors in liver cancer cells) and liver-protective effects (protecting normal liver cells through the mitochondrial-mediated apoptotic pathway) [122,123] (Figure 7). GA, a caged xanthone extracted from the *Garcinia hanburyi* tree (Figure 7), is a potent broad-spectrum anti-tumor compound (effective against lung cancer [124], breast cancer [125], gastric cancer [126], etc.), which can disrupt the REDOX homeostasis of cells (increase ROS and decrease GSH) by inhibiting mechanisms such as thioredoxin reductase (TrxR) and heat shock protein 90 (HSP90), and induce tumor cell death [127]. Inspired by the above-mentioned key points, Wang et al. [77] constructed the carrier-free GG NPs by through simple hydrophobic interaction self-assembly using the natural GA and GL with complementary biological activities. In GG NPs, GL not only serves as a building block, but more importantly, it also endows NPs with the ability to target liver cancer actively and effectively reduces the toxicity of GA to normal liver cells. This design ingeniously exploits the inherent characteristics of the two natural products, spontaneously achieving “internal synergistic enhancement” and “internal detoxification” during the formation of NPs. It does not require additional carriers or complex modifications, thus resolving the key bottlenecks of low bioavailability and high systemic toxicity faced by the clinical application of GA. The photosensitizer zinc phthalocyanine (ZnPc_2_; Figure 7) was co-self-assembled with the above-mentioned GG NPs to form a new type of carrier-free nanoparticle GGZ NPs. The assembly process significantly enhanced the photothermal effect of ZnPc_2_, greatly increasing the photothermal conversion efficiency (PCE) of GGZ NPs to up to 80.8%. In the acidic TME, the singlet oxygen (^1^O_2_) production of GGZ NPs increased by two times.

The tumor targeting of GGZ NPs is significantly better than that of free ZnPc_2_, and in vitro organ imaging confirms that its accumulation in the liver is higher. By comparing cy5-labeled GG NPs (containing GA) with GA NPs (containing GA only), GG@Cy5 NPs specifically aggregated at the liver tumor site (reaching the peak within 2 h), while GA@Cy5 NPs were dispersed throughout the body without aggregation, proving that GL endowed the liver with targeting ability. The photoacoustic imaging (PA) signal of GGZ NPs is concentration-dependent. Two hours after injection, the red signal (drug) penetrates the deepest tissue and is gradually metabolized over time. The penetration depth of free ZnPc_2_ is relatively shallow and is limited only to the epidermal area, while GGZ NPs can achieve deep delivery. Targeted accumulation enriches photothermal active substances around the tumor, achieving efficient local warming. Irradiation was performed at the peak time point of targeted accumulation (2 h). The tumor temperature in the GGZ NPs group rose to 43 °C within 5 min, which was higher than that in the control group (37 °C in the PBS group and 39 °C in the ZnPc_2_ group). The GGZ NPs + L group had the most significant tumor-suppressing effect in vivo. After 12 days, the tumor could hardly be observed with the naked eye. Targeted delivery (GA-mediated) combined with dual therapy of PTT and PDT achieves “synergistic detoxification” (external auxiliary enhancement), increasing the tumor suppression effect by 1.8 times.

HSP90 is a molecular chaperone protein that is overexpressed in tumor cells [128]. Its key role is to help cells cope with heat stress (such as the heat generated by PTT) and other environmental pressures, thereby maintaining the survival and proliferation of tumor cells. GA can specifically act on the ATP-binding domain at the N-terminal of the HSP90 protein, competitively inhibiting the activity of its chaperone protein by directly occupying the nucleotide-binding slot. Under normal circumstances, tumor cells will up-regulate HSP90 (as shown in the ZnPc_2_ + L group) during the PTT process to resist thermal and oxidative damage. When HSP90 is effectively inhibited by GA, tumor cells lose this crucial thermal protection mechanism and become more sensitive to the heat generated by PTT, thereby significantly enhancing the therapeutic effect of PTT. Compared with the control group, the use of photosensitizer ZnPc_2_ combined with laser irradiation (simulated PTT) alone significantly increased the expression of HSP90. This indicates that when tumor cells are exposed to the heat stress of PTT, they will actively up-regulate HSP90 as a protective mechanism to enhance their heat tolerance, helping the cells resist thermal and oxidative damage and thus survive. When nanoparticles containing GA were used (GG NPs treated alone or GGZ NPs treated with laser), the expression level of HSP90 was significantly decreased (the lowest when GG NPs were treated alone). This directly proves that GA effectively inhibits HSP90. The decreased expression level of HSP90 means that tumor cells have lost the key molecular chaperone protection. This weakens the cells’ ability to respond to heat stress (e.g., PTT), that is, heat tolerance decreases. Cells are unable to effectively resist the heat generated by PTT and the accompanying oxidative damage, making them more prone to damage and death. In conclusion, GA disrupts the key protective mechanism by which tumor cells rely on HSP90 to resist heat stress by specifically inhibiting the expression of HSP90 (acting on its N-terminal ATP-binding domain). In the future, through strict safety assessment and evidence-based research, the dietary components of “medicine and food homology” will play a more important role in individualized tumor treatment.

**Figure 8:**
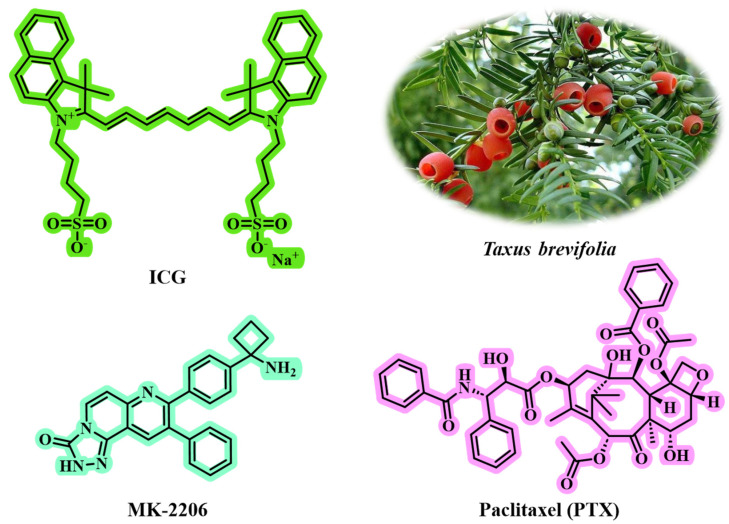
The chemical structures of ICG, MK-2206, and PTX. PTX can be isolated from the medicinal plant *Taxus brevifolia*.

Triple-negative breast cancer (TNBC) is characterized by a high recurrence rate and the poorest survival outcomes, presenting one of the most significant challenges in clinical practice [129,130]. Traditional chemotherapy has demonstrated limited therapeutic efficacy due to its non-specific biodistribution and inadequate drug accumulation [131,132]. Therefore, the development of a carrier-free nanomedicine that integrates phototherapy and chemotherapy—targeting distinct molecular pathways to enhance therapeutic effects, minimize systemic toxicity, and inhibit cancer metastasis and recurrence—is highly desirable. Wang et al. [78] successfully self-assembled the clinical indocyanine green (ICG) and PTX into carrier-free NPs (IP NPs) using a straightforward one-step nanoprecipitation method (Figure 8). This innovative approach circumvents the complexities and potential toxicity issues associated with traditional carriers, thereby improving biocompatibility and stability of the drugs. The particle size of IP NPs is affected by the mass ratio of PTX to ICG. Testing ratios of 1:2, 1:1, and 2:1 revealed that a 1:1 ratio yielded the smallest particle size (144.40 nm) and the lowest polydispersity index (PDI of 0.27), indicating it as the optimal formulation. Other ratios (1:2 or 2:1) resulted in larger sizes, measuring 153.10 nm and 194.17 nm, respectively. TEM showed that IP NPs had a uniform spherical shape with a narrow size distribution, averaging around 100 nm in size. They exhibited good stability in pure water, PBS, and RPMI-1640 medium over seven days without significant changes in particle size or PDI, demonstrating excellent long-term stability. The particle size of IP NPs increased in an acidic environment (pH 5.5), indicating pH responsiveness and promoting drug release in TME. DLS analysis revealed that the particle size and PDI of IP NPs remained stable over a 7-day period. Formed through π-π stacking, hydrophobic interactions, and electrostatic interactions, IP NPs demonstrated ultra-high drug-loading capacity and enhanced drug utilization efficiency. They maintained stable physicochemical properties across various pH levels and ionic strengths. For example, adding hydrophobic sodium dodecyl sulfate (SDS) or NaCl caused a blue shift and narrowing of the absorption peak, highlighting the role of hydrophobic and electrostatic interactions in forming IP NPs.

Flow cytometry and fluorescence microscopy showed that IP NPs were effectively taken up by 4T1 tumor cells, with uptake positively correlated to time. Under 808 nm laser irradiation, IP NPs demonstrated excellent photothermal conversion efficiency, raising the temperature to 44 °C within 2 min, sufficient to damage tumor cells. In vivo imaging indicated significant accumulation of IP NPs in tumor and longer retention compared to free ICG, highlighting good targeting and biocompatibility. IP NPs combined photothermal ICG and chemotherapeutic PTX for a dual attack on tumor cells, enhancing therapeutic effects while reducing potential MDR from single treatments. Targeted accumulation of NPs significantly increased their concentration in tumor tissues, minimizing toxicity to normal tissues and improving safety. The study introduced AKT inhibitor MK-2206, which enhanced tumor cell sensitivity to chemotherapy and phototherapy by inhibiting AKT overactivation, further decreasing tumor invasiveness. The combination of MK-2206 and IP NPs not only inhibited the AKT pathway but also impacted key signaling pathways like MAPK and NF-κB, achieving multi-pathway regulation of tumor cell survival, proliferation, migration, and invasion. In the tumor rechallenge model, this strategy effectively inhibited primary tumor growth while activating systemic anti-tumor immune responses to prevent recurrence. The biological safety of IP NPs was assessed by in vivo experiments, revealing no significant behavioral abnormalities or weight loss during treatment, and no pathological changes in major organs. The combined strategy enhanced the proportions of CD8 + and CD4 + T cells in the spleen, suggesting its potential to activate systemic anti-tumor immune responses, which is crucial for preventing tumor metastasis and recurrence. In summary, this combined treatment approach effectively inhibited tumor growth, metastasis, and recurrence, offering new ideas and strategies for clinical applications. The study systematically designed and verified all aspects from nanoparticle preparation and characterization to cell experiments and animal models while regulating signaling pathways. This ensures the reliability of the research findings and provides a solid scientific basis for developing more effective and safer cancer treatments.

The “combination” drug delivery system facilitates innovative multi-dimensional therapies by integrating chemotherapy, PTT, and PDT. This approach shows promise in overcoming various resistance forms. However, when designing multimodal treatment regimens, it is crucial to consider potential side effects of nanomedicines, including oxidative stress, inflammatory responses, and genotoxicity. Moreover, self-assembling nanostructures may pose immunogenicity risks. Further investigation into the synergistic effects of these therapies is essential, particularly regarding free radical generation, tumor targeting, and immune modulation mechanisms.

## 4. Natural Product-Based Nanomedicines for Tumor Photodynamic/Starvation Therapy

To maintain rapid proliferation, tumor cells often obtain energy and structural substances by reshaping the acquisition and utilization of glucose, amino acids, lipids, and oxygen [129,130]. The cancer starvation therapy strategy, which centers on “blocking blood supply, consuming glucose/oxygen, and other key nutrients for tumors”, has been widely studied due to its theoretical high specificity and potential broad-spectrum indications [131,132]. This strategy emphasizes intervening in the TME and metabolic pathways to weaken the energy production and biosynthesis capabilities of tumor cells, thereby inhibiting growth, promoting apoptosis or enhancing sensitivity to other treatments.

**Figure 9:**
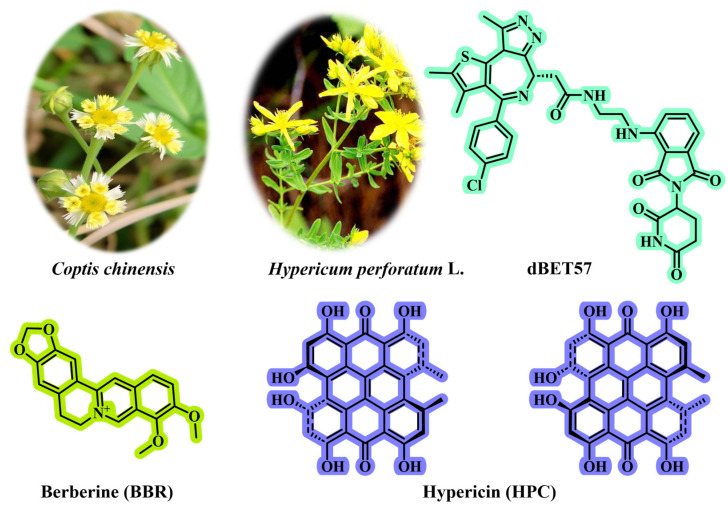
The chemical structures of BBR, HPC, and dBET57. BBR can be isolated from the medicinal plant *Coptis chinensis*; HPC can be isolated from the medicinal plant *Hypericum perforatum* L.

Intrigued by this motivation, Zhang et al. [79] developed a dual-targeting nanoplatform (BHP NPs) for tumor photodynamic/starvation therapy. BHP NPs are directly self-assembled from three active molecules (BBR, HPC, and dBET57; Figure 9) via the nanoprecipitation method without needing additional synthetic carriers (such as DSPE-PEG or PLGA), fundamentally avoiding the issue of carrier materials. The structural skeletons of natural products (such as BBR and HPC) not only possess biological activity but also have self-assembly capabilities. BBR, HPC, and dBET57 spontaneously aggregate through non-covalent interactions. Due to the pure drug self-assembly mechanism, BHP NPs achieve a drug-loading efficiency of 92%, significantly increasing the drug-loading capacity. The self-assembled BHP NPs achieve dual targeting (mitochondrial targeting + necrotic region targeting) through the following mechanisms, significantly enhancing tumor penetration and enrichment.

BBR is a positively charged alkaloid that can specifically target the negatively charged mitochondrial membrane through electrostatic interactions. This targeting enables BHP NPs to efficiently co-deliver PROTAC (dBET57) and photosensitizer (HPC) to the “energy factory” of tumor cells—mitochondria. This directly leads to precise energy strikes, with dBET57 degrading BRD4 protein (inhibiting key glycolysis factors such as c-Myc, etc.), and BBR itself inhibiting mitochondrial respiratory complex I, simultaneously blocking both glycolysis and oxidative phosphorylation (OXPHOS) energy metabolic pathways, causing ATP production to drop by 85%, achieving a more thorough “starvation therapy” and overcoming the compensatory problem of OXPHOS caused by single glycolysis inhibition. Energy deprivation (especially OXPHOS inhibition) itself consumes less oxygen and may improve tumor hypoxia, indirectly benefiting traditional oxygen-dependent PDT. However, more importantly, light-triggered OXPHOS blockage provides a direct energy strike method that does not rely on oxygen. dBET57-mediated PD-L1 targeted degradation offsets the possible upregulation of PD-L1 induced by PDT, ensuring that the PDT-induced immunogenic cell death (ICD) effect can effectively activate anti-tumor immunity.

Self-assembled NPs retain the core characteristics of HPC, a necrosis-affinity photosensitizer that binds explicitly to subcellular substances exposed in necrotic tissues, such as degraded proteins, peptides, and nucleotides. This property spatially overlaps with the pathological features of the TME, which often contains necrotic regions within solid tumors, endowing BHP NPs with unique deep-penetrating capabilities. This enables BHP NPs to actively target and accumulate in the existing necrotic regions of tumors. During PDT treatment, the ROS generated by HPC can induce more tumor cell death/necrosis, and the newly formed necrotic regions will further attract more BHP NPs (containing HPC) to aggregate in deeper areas. Experiments have demonstrated that BHP NPs can penetrate over 80 μm in 3D tumor spheroids and up to 1600 μm in tumor tissues in vivo. This is far superior to PEGylated HPC@PEG NPs (with a penetration depth of <800 μm) or NPs using the non-targeted dye DiR instead of HPC (DiR@BP NPs). This deep-penetrating ability overcomes the limitations of traditional nanomedicines (relying on passive EPR effect) in delivering drugs through complex TME biological barriers, such as dense matrix and high interstitial pressure, ensuring that drugs can reach deep tumor cells. The inherent fluorescence property of HPC enables the real-time monitoring of the in vivo distribution of BHP NPs. Studies have found that their accumulation at the tumor site reaches a peak 12 h after injection, providing visual guidance for precisely selecting the optimal light treatment time window, maximizing therapeutic efficacy and reducing off-target effects.

In summary, this study creatively designed and constructed a carrier-free mitochondrial-targeted nanoplatform (BHP NPs) based on natural product self-assembly: (1) Simplified preparation through highly efficient self-assembly and avoided carrier toxicity; (2) Achieved a “metabolic blockade” at the subcellular organelle (mitochondria) level by PROTAC-mediated glycolysis inhibition and natural product-mediated OXPHOS inhibition; (3) Established a positive feedback loop using the necrosis affinity of photosensitizers to achieve deep tumor penetration; (4) Synergistically enhanced the PDT destructive power and triggered ferroptosis via mitochondrial co-localization; (5) Utilized intrinsic fluorescence to visualize and precisely control the treatment process. These characteristics collectively significantly enhanced the accumulation, penetration, and comprehensive therapeutic efficacy (synergistic photodynamic/hypoxia/immunotherapy) of BHP NPs in complex TNBC models. Moreover, without a carrier, it has better biocompatibility and potential to reduce systemic toxicity, providing an innovative and efficient solution to overcome the challenges of existing PROTAC-PDT combination therapy (low drug loading, shallow penetration, metabolic compensation, and immunosuppression).

The multifunctional nanoplatform based on BBR and HPC can be efficiently prepared through supramolecular self-assembly, avoiding complex chemical reactions. This method addresses preparation challenges in nanomedicine and improves biocompatibility issues. Although BHP NPs, as carrier-free nano-systems, are designed to enhance tumor penetration and accumulation, their actual penetration depth and uniform distribution in complex TME still need to be further verified in models closer to clinical settings, such as patient-derived xenograft models. Although BHP NPs are self-assembled from natural products with the aim of reducing toxicity, the systemic safety of long-term use and potential impacts on normal tissues, especially high-metabolic organs, still require more in-depth assessment. Although the integration of “starvation therapy” aims to alleviate hypoxia by reducing the oxygen consumption of tumor cells, in the core areas of severe hypoxia/necrosis of tumors, it may be difficult to effectively generate ROS due to extreme hypoxia, which may limit the efficacy of PDT. The high heterogeneity of TNBC means that responses may vary significantly among different subtypes or individual patients. The efficacy and safety of BHP NPs in a broader range of preclinical models, such as PDX models of different subtypes, and ultimately in humans, still need to be further validated. It is important to establish a standardized evaluation framework and long-term monitoring mechanisms to enhance the clinical translation of nanoplatforms. For example, assessment criteria should address physicochemical properties, biocompatibility, and potential toxicity of nanomaterials. Additionally, long-term follow-up studies are essential to evaluate the health impacts of nanodrugs, including immune responses, organ function, and tumor recurrence rates.

## 5. Natural Product-Based Nanomedicines for Tumor Bioimaging and PDT-Chemotherapy

Early diagnosis of diseases should effectively leverage bioimaging techniques [133,134]. Non-invasive bioimaging methods typically necessitate the use of specific probes to monitor and quantify biological pathways in living systems [135]. These imaging agents must possess the following characteristics: (1) a significant contrast effect, which entails a high signal-to-noise ratio under physiological conditions; (2) stability against the action of various enzymes or proteases present in serum or target tissues within the body, along with rapid clearance by healthy organs; and (3) cost-effectiveness coupled with environmentally sustainable production practices.

Active targeting is a strategic approach that facilitates the selective recognition of tumor cells or the TME, enhances drug uptake, and improves therapeutic efficacy by modifying specific biological target ligands on the surface of nano-delivery platforms [136,137]. In contrast to passive targeting, active targeting focuses on the differential expression of target molecules, receptor-mediated endocytosis pathways, and the regulation of local drug aggregation and distribution at tumor sites. Lactose, a disaccharide composed of galactose and glucose, can specifically bind to the highly expressed asialoglycoprotein receptor (ASGPR) present on liver cancer cells (such as HepG2), thereby achieving targeted delivery [138,139]. Compared to nano-delivery systems that rely on the EPR effect associated with passive targeting, lactose-mediated active targeting increases drug enrichment efficiency while minimizing ineffective loss in physiological environments.

**Figure 10:**
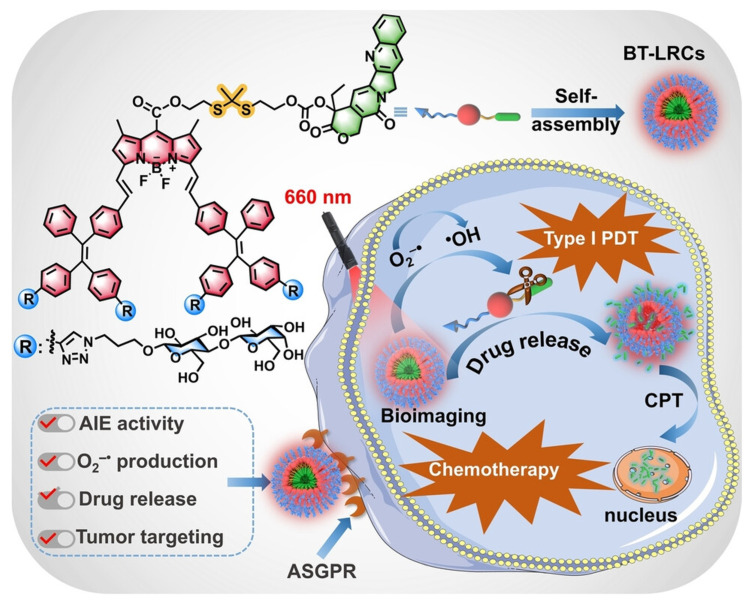
Schematic illustration of BT-LRCs for tumor bioimaging and PDT-chemotherapy. Reproduced from Ref. [80] with permission from © 2024 Wiley-VCH.

Zhou et al. [80] creatively integrated the Type I PDT mechanism, AIE characteristics, precise targeting of glycosylation (ASGPR recognition + hydrogen bond + regulation of aggregation/ROS), and ROS-responsive drug delivery (thioacetone bond) into a nanoplatform (BT-LRCs) efficient and visualized photocontrolled synergistic therapy (Type I PDT + chemotherapy) in the hypoxic TME (Figure 10). Firstly, tetraphenylethylene (TPE, AIE unit) was introduced into the BODIPY (photosensitizer) structure to create a novel aggregation-induced luminescence (AIE) active photosensitizer system (BT-LRC). This design overcomes the agglomeration-induced quenching (ACQ) problem caused by π-π stacking in the aggregated state of traditional photosensitizers. BT-LRC exhibits intense red fluorescence emission in the aggregated state (with a significant increase in fluorescence quantum yield), which endows it with both highly efficient photodynamic effects and excellent fluorescence imaging capabilities, making it suitable for visualization-guided therapy. CPT was linked to the glycosylated photosensitizer with AIE activity through a ROS-cleavable thioketal bond to form the prodrug BT-LRCs. This strategy improved the problems of poor water solubility, unstable lactone ring, and non-specific toxicity of CPT. Lactose was introduced as the targeted ligand on the photosensitizer–prodrug conjugate (BT-LRCs). Lactose can specifically recognize the ASGPR highly expressed on the surface of HepG2, achieving active targeting and enhancing the enrichment of drugs at tumor sites. In addition, lactose can interact with the cell surface through hydrogen bonds to further enhance the targeting effect.

Hydrophilic lactose units profoundly regulate the aggregation behavior of BT-LRCs in aqueous solutions. Unlike non-glycosylated BT-RCs, which exhibit ACQ, BT-LRCs exhibit significant AIE characteristics. Theoretical calculations (DFT/RDG) indicate that the abundant hydrogen bonds and van der Waals forces formed between lactose units stabilize the molecular structure, restrict intramolecular rotation/vibration, while the distorted molecular conformation and steric hindrance effectively inhibit π-π stacking. This is a key advantage unexpectedly brought by glycosylation modification, which solves the ACQ problem and enhances the ability to produce fluorescence and ROS. The sugar–sugar interaction between lactose units in the aggregated state has been proven to be one of the key reasons why BT-LRCs have an outstanding ability to generate ROS.

This nanoplatform has achieved the synergy of PDT and chemotherapy. Experiments have fully demonstrated that BT-LRCs have an outstanding synergistic anti-tumor effect under red light irradiation, and its efficacy is significantly superior to that of single therapy (PDT alone or chemotherapy alone) or non-targeted/non-glycosylated control groups (BT-LCCs, free CPT). The tumor mass in the BT-LRCs treatment group was much lower than that in the control group, and it induced the most significant apoptosis and necrosis of tumor cells, with high safety. In terms of mechanism, under red light (660 nm) irradiation, BT-LRCs generate a large amount of ROS at the tumor site (through ASGPR targeted enrichment): (1) Directly cleaving the thioacetone bond to release CPT, exerting a chemotherapy effect; (2) ROS itself causes oxidative damage to tumor cells. CPT itself has the effect of inhibiting HIF-1α, which may make tumor cells more vulnerable in a hypoxic environment. Red light irradiation provides a remote spatio-temporal control of drug release switch. The AIE fluorescence characteristic of BT-LRCs itself enables it to be used for real-time and precise imaging to monitor drug distribution and regulate the release process (visualized synergistic therapy).

The use of multiple therapeutic agents requires a delivery platform that standardizes pharmacokinetics and pharmacodynamics, extends circulation time, selectively targets sites, and enables controlled release. To achieve this, therapies should synergistically target distinct but interconnected oncogenic signaling pathways. Additionally, the design of combination drug delivery platforms must not complicate production or quality control processes. During development, it is crucial to consider factors such as chemical synthesis capabilities and industrial-scale manufacturing needs (including batch consistency, scalability, material stability, economic feasibility, and self-assembly characteristics) for successful clinical translation.

## 6. Natural Product-Based Emerging Nanomedicines for Tumor Chemo-Radio-Theranostics

Radiopharmaceutical therapy (RPT) is a form of targeted radiation treatment in which radioactive compounds are specifically designed to seek out cancer cells or tumor-associated targets, delivering cytotoxic radiation from within or nearby [140,141]. By combining molecular targeting with radiative damage, RPT aims to maximize the destruction of tumors while minimizing exposure to normal tissues [142,143]. Chemo-radio-thanostics integrates diagnostic precision, targeted radiopharmacology, and systemic chemotherapy in a closed loop of “diagnosis-treatment-assessment”. It utilizes molecular imaging to clarify tumor characteristics and burden. Targeted radiopharmaceuticals deliver specific internal radiation therapy, while systemic chemotherapy targets micrometastatic lesions to enhance radiosensitivity. At the core of this approach are diagnostic precision and targeted radiopharmacology. Commonly used diagnostics like Ga-68 and F-18 labeled PET, along with SPECT/CT, help select suitable patients for targeted therapies, localize lesions, and assess tumor heterogeneity [144,145]. The therapeutic aspect leverages radioactive isotope carriers; β-emitting isotopes (e.g., Lu-177) enable irradiation, while α-emitting isotopes (e.g., Ac-225) provide high linear energy transfer for precise destruction [146,147]. Integration with systemic chemotherapy offers unique advantages by addressing micrometastases or unknown lesions while sensitizing tumors to radiation through enhanced sensitivity. Additionally, it may yield benefits from combined therapies by altering the immune microenvironment.

**Figure 11:**
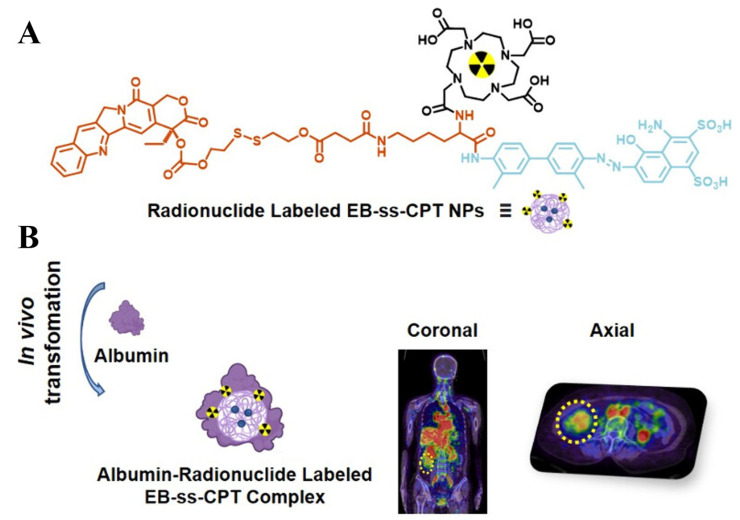
Schematic illustration of DOTA-EB-ss-CPT NPs for tumor chemo-radio-theranostics. (**A**) Chemical structure of DOTA-EB-ss-CPT; (**B**) Anti-tumor effects of DOTA-EB-ss-CPT NPs. Reproduced from Ref. [81] with permission from © 2025 American Chemical Society.

Xiong et al. [81] designed and synthesized an Evans blue conjugated CPT nanoprodrug (EB-CPT) modified with DOTA or NOTA (Figure 11). This molecule can self-assemble into NPs, which can be effectively labeled with therapeutic radionuclides thulium-177 (^177^Lu) or diagnostic radionuclides gallium-68 (^68^Ga)/copper-64 (^64^Cu). The core of nanomedicines preparation lies in achieving self-assembly via a one-step nanoprecipitation method: the prodrug DOTA-EB-ss-CPT spontaneously forms NPs during the solvent exchange process, and then methanol is removed to stabilize the structure. This method benefits from the interaction between its hydrophobic and hydrophilic parts, requiring no additional carriers, and being simple and efficient. The average hydrodynamic diameter (HD) was measured by DLS to be 98.7 nm. Transmission electron microscopy (TEM) images show that they are uniform spherical nanoparticles, and their size is consistent with the DLS results. After being stored at room temperature in PBS for 19 days, DOTA-EB-ss-CPT NPs remained stable through continuous DLS measurements, and no significant size changes. Similarly, during the 21-day storage period in the serum solution, no significant changes in the HD of DOTA-EB-ss-CPT were observed. Albumin is the most abundant protein in plasma, with an extremely long circulation characteristic and the ability to accumulate preferentially in tumor tissues through EPR effects. DOTA-EB-ss-CPT has the characteristic of high affinity binding with serum albumin (apparent dissociation constant Kd = 4.50 × 10^−6^ M), forming stable albumin/drug nanocomplexes. Fluorescence kinetics studies showed that after the addition of albumin, the EB fluorescence of DOTA-EB-ss-CPT was significantly enhanced (up to 7.1 times), indicating the formation of a stable complex. Albumin “free-rider” mechanism enhances targeting: Through the specific binding of EB to blood albumin, the blood circulation time of NPs is significantly prolonged, and the EPR effect in tumor tissues is utilized to enhance the accumulation of drugs at tumor sites.

In the mouse model of colorectal cancer (HCT116), a single injection of [^177^Lu]Lu-DOTA-EB-CPT combined with EB-CPT significantly inhibited tumor growth, and the effect was superior to that of single therapy. ^64^Cu-labeled NPs have an extended retention time in tumors (>72 h), and precise imaging can be achieved through PET/CT. The first human study (^68^Ga labeling) confirmed that it was well tolerated in patients. [^68^Ga]Ga-NOTA-EB-ss-CPT can be effectively enriched in tumor lesions, and its uptake continuously increases over time. In the late stage after administration (4 h), it is higher than that in the surrounding normal tissues, thereby providing contrast for PET/CT imaging and making it a promising diagnostic PET tracer. In summary, the core breakthrough of this research lies in integrating chemotherapy drugs, radionuclide carriers, and albumin-targeting EB into a single self-assembly unit through molecular design, which has solved the problem of pharmacokinetic mismatch in traditional combination therapies, and achieved the integration of diagnosis and treatment, providing a new path for clinical transformation.

Integrating multimodal analysis techniques can yield collaborative insights from diverse biological datasets, driving innovation in nanomedicine. For example, multi-omics data (genomics, proteomics, metabolomics) from patient-derived xenografts can train machine learning models to predict which tumor subtype will best respond. This advancement allows for precise patient classification based on radiological evidence, clarifies the pharmacokinetics of nanoscale therapeutic drugs, guides personalized intervention plans—including optimal dosing regimens and targeted delivery mechanisms—and supports comprehensive treatment evaluation. Looking ahead, individualized dosimetry, AI-assisted imaging analysis and dose prediction, as well as the development of new target-targeted drugs, will continue to expand the indication spectrum and therapeutic depth. Integrated research on combined immunotherapy, external radiotherapy and local treatment strategies is expected to enhance the synergy between local control and systemic control. AI algorithms have enhanced image analysis by improving resolution and identifying subtle diagnostic patterns [148]. Overall, chemo-radio-thanostics represents a modern multimodal treatment paradigm that takes diagnostic accuracy as the premise, targeted radiopharmacology as the core, and systemic chemotherapy as the support.

## 7. Natural Product-Based Carrier-Free Nanomedicines for Tumor GT/PTT/Chemotherapy

GT represents a promising therapeutic approach [149]. In comparison to small molecule targeted drugs, GT often offers enhanced controllability of the “therapeutic window” and possesses multi-target regulatory capabilities [150]. This is advantageous in addressing MDR and modifying TME [151,152]. GT has the potential to achieve tumor selectivity while minimizing non-specific damage by intervening in the expression, repair, or editing of tumor-associated genes [153]. The integration of GT presents advantages that singular treatments cannot achieve, such as the prevention of tumor metastasis and recurrence.

**Figure 12:**
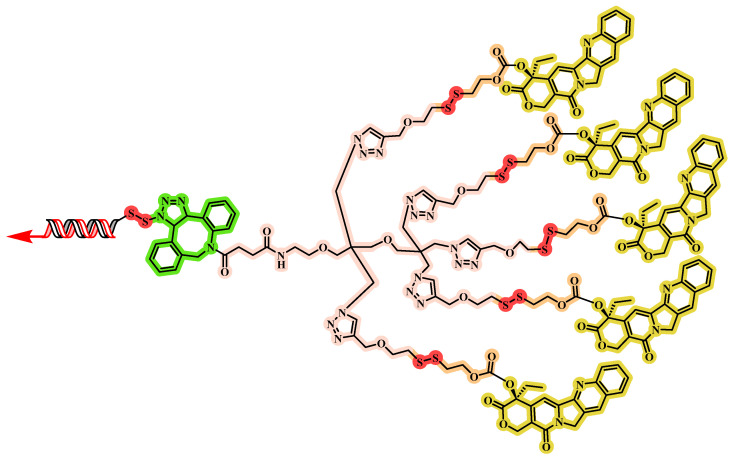
The chemical structure of Apt-5CPT.

Wang and colleagues [82] engineered a carrier-free DNA nanostructure through targeted chemical modification and orchestrated nucleic acid self-assembly (Figure 12). Five CPT units were attached to a branched scaffold with an additional arm for DNA aptamer conjugation, yielding Apt-5CPT. In parallel, the photosensitizer HPPH was covalently linked to antisense oligonucleotides to form an amphiphilic monomer, HPPH-AS. The modified DNAs Apt-5CPT and HPPH-AS were combined in a 1:5 molar ratio in DMSO and gradually introduced into deionized water under stirring, followed by dialysis and ultrafiltration to produce the multifunctional Apt-CHA. This self-assembly enables co-loading of multiple therapeutics and tumor targeting via DNA aptamers. Apt-CHA was characterized by an approximate size of 50 nm by DLS, and TEM confirmed a spherical, uniform morphology. This nanoscale size supports in vivo stability and enables passive tumor targeting via the EPR effect, potentially enhancing tissue penetration and cellular uptake. Stability assessments revealed a low CMC of 116.6 nM, indicating robust self-assembly at low concentrations. Size remained stable in both deionized water and simulated body fluid, with no significant aggregation or dissociation observed. The surface potential was about −18 mV, which helps minimize non-specific adsorption and further improves colloidal stability. Collectively, these properties ensure structural and functional integrity for effective tumor therapy.

In the PC-3 xenograft model, Apt-CHA + L (laser irradiation) produced the greatest tumor suppression, with significantly lower tumor volumes and weights than all other groups. Apt-CHA + L also markedly downregulated HSP27 mRNA and protein in tumor tissues, indicating effective GT. H&E and TUNEL analyses revealed the highest levels of tumor cell apoptosis in the Apt-CHA + L group. H&E staining of major organs showed no obvious systemic toxicity at the tested dose for the uncoated DNA nanostructure. Immunofluorescence imaging demonstrated that Apt-CHA could extravasate from tumor vessels and penetrate deeply into the tumor. Overall, the tri-combination treatment with Apt-CHA + L achieved substantial tumor growth inhibition and increased apoptosis, highlighting its potential for personalized cancer therapy. Its modular, scalable design allows exchanging or adding components (such as gene-editing tools or protein drugs) for personalized cancer therapy, indicating broad clinical translation potential.

However, the integration of GT with nanoplatforms still faces numerous challenges. Firstly, a systematic investigation into the metabolic and toxicological characteristics is essential. Secondly, there exists significant potential for enhancing gene transfer efficiency. Furthermore, evaluating the translational efficacy of gene therapeutic agents is crucial for determining appropriate dosing regimens and drug ratios within delivery systems. The future development trend emphasizes the synergistic effect of multimodal therapy. A further understanding of the driver gene network and the immune–tumor interaction will help develop more efficient treatment regimens. At the same time, cost and accessibility also need to be fully considered in clinical translation to achieve extensive clinical application and public health benefits. Overall, although malignant tumors still pose a global health burden, with the deepening of basic research and the advancement of clinical treatment strategies, the prospects for GT are gradually being realized, and the quality of life and survival rate of patients are expected to continue to improve.

## 8. Challenges and Perspectives

The emergence of carrier-free “triadic” drug delivery platforms derived from natural products represents a novel paradigm in cancer treatment, owing to their remarkable capabilities. These platforms are characterized by the ability to integrate multiple action mechanisms, enhance drug targeting, and incorporate various agents. However, several key challenges must be addressed.

Challenges and Bottlenecks. Firstly, batch-to-batch consistency and reproducibility are key issues in pharmaceutical-grade applications. Secondly, particle size distribution, stability, and drug release mechanisms need systematic optimization to ensure a controllable release rate in vivo. The size of nanoparticles influences in vivo behavior following intravenous injection, affecting blood circulation, accumulation, and retention within tumors. Particles exceeding 100 nm are typically sequestered by organs such as the liver and kidneys due to high blood flow rates and a substantial presence of mononuclear macrophages; these particles are subsequently eliminated from the body through metabolic processes [154]. Consequently, there is an urgent need for guidelines on the preparation of carrier-free nanomedicines aimed at optimizing particle size for effective in vivo delivery. At the same time, certain carrier-free “triadic” drug delivery platforms assembled through non-covalent interactions can maintain structural integrity; however, this stability may be compromised under physiological conditions. Such instability could lead to premature drug release during systemic circulation, potentially reducing the efficacy of tumor-specific drugs and increasing off-target toxicity risks. Additionally, scale-up production, quality control, and regulatory compliance are also significant obstacles. Safety assessment should cover long-term exposure, immune responses, and the potential effects of metabolites. Evaluation strategies and research priorities include: characterization of particle size, morphology, and distribution (DLS, TEM/SEM), stability assessment, drug loading and release profiles, in vivo pharmacokinetics and distribution, tumor enrichment and penetration ability, cellular uptake pathways and cytotoxicity, as well as in vivo efficacy and safety. Experimental designs should closely simulate clinical scenarios, such as stability in complex blood environments, therapeutic effects in various tumor models, and comparative advantages over existing drugs. Standardized preparation and characterization procedures are crucial for cross-study comparisons.

Future Development Directions: (1) Multidrug self-assembly and molecular-level regulation: Through drug–drug synergistic design, achieve multidrug synergy while maintaining high drug loading. (2) Environment-responsive release: Design intelligent self-assembly systems based on tumor-specific features such as low pH and high reductivity to achieve precise drug release. (3) Integration with diagnosis and treatment: Combine imaging drugs or photothermal, photodynamic, and other auxiliary treatments to build an integrated therapeutic platform. (4) Quality standards and regulatory pathways: Establish standardized preparation, characterization, and quality control systems to facilitate clinical translation. (5) Biological mechanism research: Deepen understanding of the in vivo behavior, immunological impact, and MDR mechanisms of NPs.

To enhance clinical translation, the design of natural product-based “triadic” nanoplatforms should be both reliable and straightforward, minimizing unnecessary complexity. This carrier-free approach effectively addresses common limitations associated with small molecule drugs—such as poor targeting and low bioavailability—while also overcoming challenges related to inadequate preparation and biocompatibility in certain nanomedicines. To advance natural product-based nanoplatforms for effective clinical application, it is essential to implement innovative strategies that fully leverage their potential, particularly in terms of safety and clinical efficacy. It is important to emphasize that the clinical transformation of natural product-based, carrier-free, “triadic” drug delivery platforms necessitates collaboration across various disciplines. Recent advancements in artificial intelligence, particularly in language processing and innovative frameworks, have created transformative opportunities [155,156]. Machine learning algorithms are revolutionizing therapeutic innovation through comprehensive health data analysis, precise biomarker identification, and enhanced anomaly detection. AI-driven algorithms significantly improve image analysis by increasing resolution and identifying subtle diagnostic patterns. This progress enables accurate patient classification based on imaging evidence, elucidates the pharmacokinetic characteristics of nanoscale therapies, and facilitates the development of personalized intervention plans—including optimal dosing regimens and targeted delivery mechanisms—while promoting thorough treatment evaluation.

In the context of “regulatory obstacles and scalability of nanomedicines”, regulatory pathways are significantly influenced by structural complexity and biological behaviors. It is crucial to establish verifiable quality attributes and drug release mechanisms at an early stage. Developing consistent characterization methods that prioritize recognized measurement indicators and control standards is essential. The controllability of the amplification process relies on stable control measures and traceability. It is important to anticipate potential regulatory objections and document response strategies to ensure effective change management traceability, thereby mitigating risks associated with batch-to-batch variability. Integrating regulatory requirements during the design phase minimizes risks and costs related to subsequent changes, thereby enhancing the likelihood of successful registration. Regulatory disparities necessitate that research teams develop a unified framework for global development while ensuring compliance strategies tailored to specific regions. Core evidence should encompass safety data (including immunogenicity, chronic toxicity, and tissue accumulation) in conjunction with efficacy data. Establishing an interdisciplinary compliance communication mechanism is vital for ensuring cohesive collaboration among R&D, production, regulatory affairs, and clinical teams.

In sum, carrier-free “triadic” drug delivery platforms derived from natural products, with the drug itself as the core component, demonstrate the potential advantages of “reduced carrier dependence and high drug efficacy expression”, providing a theoretical basis for enhancing the therapeutic window, reducing toxicity, and simplifying preparation [157]. Despite challenges in reproducibility, scale-up production, and safety, with the maturation of design concepts, advancements in characterization techniques, and the improvement of regulatory frameworks, this field is expected to leap basic research to clinical application in the future, offering novel, economical, and efficient drug delivery strategies for cancer treatment.

## Figures and Tables

**Table 1:** Natural product-based carrier-free “triadic” drug delivery platforms for enhanced tumor treatment (2024–2025).

Natural Products	Therapeutic Agents	Size [nm]	Therapy Methods	Results	Refs.
Camptothecin	DTX@ERL-SS-CPT NPs	121.0	Combinational chemotherapy	Mitigating multidrug resistance in lung cancer while improving drug delivery efficiency; real-time monitoring of drug release	[76]
Camptothecin, curcumin, and erianin	CUR@CPT-SS-ERI NPs	162.0	Combinational chemotherapy	Enhancing drug delivery efficiency and mitigating multidrug resistance in lung cancer	[76]
Gambogic acid and glycyrrhizic acid	GGZ NPs	233.2	PDT/PTT/chemotherapy	Dual ‘synergy and attenuation’ for improved liver cancer phototherapy	[77]
Paclitaxel	IP NPs	144.4	PDT/PTT/chemotherapy	Combined therapy to suppress tumor growth, metastasis, and recurrence	[78]
Berberine and Hypericin	BHP NPs	111.5	Photodynamic/starvation therapy	Target tumor cells via dual action on mitochondria and necrotic cells to improve penetration and effectiveness	[79]
Camptothecin	BT-LRCs	~90	Bioimaging and PDT-chemotherapy	Enhanced therapeutic effect in HepG2 cells and tumor-bearing mice	[80]
Camptothecin	DOTA-EB-ss-CPT NPs	98.7	Chemo-radio-theranostics	Achieving a balance among tumor accumulation, safety, and diagnostic efficacy	[81]
Camptothecin	Apt-CHA	~50	GT/PTT/chemotherapy	Combination of chemotherapy, PDT, and GT for personalized cancer treatment	[82]

## Data Availability

No new data were created or analyzed in this study. Data sharing is not applicable to this article.

## Abbreviations

AIE	aggregation-induced luminescence
ASGPR	asialoglycoprotein receptor
BBR	berberine
CPT	camptothecin
CUR	curcumin
ERI	erianin
FDA	Food and Drug Administration
GA	gambogic acid
GL	glycyrrhizic acid
GSH	glutathione
GT	gene therapy
HPC	hypericin
ICD	immunogenic cell death
ICG	indocyanine green
LAC	lactose
MDR	multidrug resistance
PA	photoacoustic imaging
PDT	photodynamic therapy
P-gp	P-glycoprotein
PS	photosensitizer
PTT	photothermal therapy
PTX	paclitaxel
ROS	reactive oxygen species
TNBC	triple-negative breast cancer
TrxR	thioredoxin reductase
TME	tumor microenvironment
